# Male Use of Female Sex Work in India: A Nationally Representative Behavioural Survey

**DOI:** 10.1371/journal.pone.0022704

**Published:** 2011-07-29

**Authors:** Michelle F. Gaffey, Srinivasan Venkatesh, Neeraj Dhingra, Ajay Khera, Rajesh Kumar, Paul Arora, Nico Nagelkerke, Prabhat Jha

**Affiliations:** 1 Centre for Global Health Research, Li Ka Shing Knowledge Institute, St. Michael's Hospital and Dalla Lana School of Public Health, University of Toronto, Toronto, Ontario, Canada; 2 National AIDS Control Organisation, New Delhi, India; 3 Ministry of Health and Family Welfare, New Delhi, India; 4 School of Public Health, Postgraduate Institute of Medical Education and Research, Chandigarh, India; University of Cape Town, South Africa

## Abstract

Heterosexual transmission of HIV in India is driven by the male use of female sex workers (FSW), but few studies have examined the factors associated with using FSW. This nationally representative study examined the prevalence and correlates of FSW use among 31,040 men aged 15–49 years in India in 2006. Nationally, about 4% of men used FSW in the previous year, representing about 8.5 million FSW clients. Unmarried men were far more likely than married men to use FSW overall (PR = 8.0), but less likely than married men to use FSW among those reporting at least one non-regular partner (PR = 0.8). More than half of all FSW clients were married. FSW use was higher among men in the high-HIV states than in the low-HIV states (PR = 2.7), and half of all FSW clients lived in the high-HIV states. The risk of FSW use rose sharply with increasing number of non-regular partners in the past year. Given the large number of men using FSW, interventions for the much smaller number of FSW remains the most efficient strategy for curbing heterosexual HIV transmission in India.

## Introduction

India had about 1.4 to 1.6 million people living with HIV in 2006 [1]. National HIV prevalence at ages 15–49 years is about 0.25–0.28% [1], but varies by region. The four high-HIV prevalence southern states of Andhra Pradesh, Karnataka, Maharashtra and Tamil Nadu account for only 30% of India's population but about 64% of its HIV infections [2].

HIV transmission in India occurs primarily through heterosexual contact [3], with most of it driven by the male use of female sex work [4]–[6]. As in other Asian settings, the spread of HIV in India, including into the general population, depends largely on the size of the female sex worker (FSW) and client populations and on the rate of their unprotected sexual contact [7].

Previous studies in India have investigated HIV risk behaviour among FSW [8]–[12] but few studies have examined FSW use among Indian men. Prior research on factors associated with FSW use in India has been limited to high-risk male subpopulations, such as urban homeless men [13], urban sexually transmitted infection (STI) clinic attendees [14], or rural voluntary counselling and testing (VCT) clinic attendees [15], and to general population-based studies in small geographical areas [16], [17]. Here we report a nationally representative study on the prevalence and correlates of having any non-regular sex partners (NRP) and of using FSW among men aged 15–49 years in the Indian general population. We also estimate the absolute number of men who used FSW in India in 2006.

## Methods

### Ethics statement

Participants in Behavioural Surveillance Surveys conducted by India's National AIDS Control Organisation (NACO) provided informed consent verbally, documented by interviewer signature [18]. This secondary analysis of these data was approved by the ethics review board of St Michael's Hospital, Toronto, Ontario, Canada.

### Study population

NACO conducted national surveys of sexual behaviour in 2001 and 2006. The sampling and data collection methods are published [18], [19]. Multi-stage, stratified cluster sampling was used to select a nationally representative probability sample of men and women aged 15–49 years in the general population. In the 2006 round, trained interviewers administered questionnaires in the household to 48 623 men and 48 617 women.

Of the 48 623 men interviewed nationally, we excluded 5417 men from the seven northeastern states (i.e. Arunachal Pradesh, Manipur, Meghalaya, Mizoram, Nagaland, Sikkim and Tripura; about 4% of the national adult male population) where HIV transmission is driven largely by injection drug use [20]. Of the 43 206 men in the remaining 28 states, we excluded 11 936 men who reported no lifetime sexual activity and 230 sexually active men for whom information on NRP in the past year was missing, leaving 31 040 men in the study population. Andhra Pradesh, Karnataka, Maharashtra and Tamil Nadu comprise the four “high-HIV” states and the remaining 24 states comprise the “low-HIV” states, based on HIV prevalence among pregnant women aged 15–24 years in 2001–07 [21], [22].

### Study variables

Two self-reported outcomes in the previous year were examined: (i) any NRP, and (ii) use of FSW. Socio-demographic characteristics included age, urban residency, level of education and employment in the transport sector. Indicators of knowledge and awareness about HIV/AIDS and STI included awareness of a local HIV testing centre, having heard of STI other than HIV, receiving any interpersonal education about STI or HIV/AIDS in the past year, and having correct knowledge about HIV prevention and transmission. Men were assessed as receiving interpersonal education if they answered positively to either of two questionnaire items: “Did anyone in the past one year approach you to educate you on the spread of STI/HIV/AIDS?” and “Did anyone in the past one year approach you to educate you on use of condoms to prevent STI/HIV/AIDS?” Correct knowledge about HIV prevention and transmission was a composite indicator [23] that included the identification of two primary prevention methods (i.e. having one uninfected, monogamous partner; and correct, consistent condom use) and the rejection of three myths about transmission (i.e. HIV can be transmitted by mosquito bites or through sharing food with an HIV-infected person, and cannot be transmitted from a healthy-looking person). Sexual behaviour indicators included sexual debut before the age of 17 years, self-reported genital discharge or ulcer/sore in the past year, number of NRP in the past year, consistency of condom use with all NRP, and consistency of condom use with wife. Consistent condom use was defined as “always” using condoms in the past year versus “sometimes” or “never” using condoms.

### Data analysis

Analyses were conducted using national sampling weights calculated by NACO to adjust for sex ratio and urban/rural sampling proportion in each state [18]. Prevalences of NRP and of using FSW were stratified by HIV region and marital status, and standardized to the age distribution of all unmarried, married and previously married men in the study population. The analyses of factors associated with using FSW were restricted to men reporting at least one NRP in the past year. Univariate associations between FSW use and explanatory variables were first assessed by Chi-squared tests; a backward stepwise approach was then used to generate multivariate Poisson regression models to estimate adjusted prevalence ratios. Estimates for the absolute number of FSW clients in 2006 were calculated by applying the stratum-specific sample prevalences of FSW use to the projected 2006 male population in each stratum. The male population for each stratum was derived by combining Indian census projections of the 2006 male population by age and state [24] with Sample Registration System data on the age and marital status distributions of the 2006 male population [25]. Excess risk of using FSW due to having two or more NRP versus one NRP was calculated using the standard formula for attributable fraction (i.e. (RR-1)/RR, where RR is the relative risk of using FSW).

## Results

### Prevalence of having non-regular partners and of using female sex work

The survey captured information on previous year sexual partnerships for 5263 and 25 777 sexually active men aged 15–49 years in the high-HIV and low-HIV states respectively, of which 4730 (88%) men and 22 712 (87%) men were married. Among all 31 040 men (unmarried, married or previously married), 3423 men (age standardized prevalence 11.8%; 95%CI 11.4–12.2) reported at least one NRP in the past year and 1138 (3.6%; 3.4–3.9) reported at least one FSW partner (Table 1). Stratified by marital status, prevalences of NRP and of FSW use in the high-HIV states were 1.5 to 3.5 times as high as in the low-HIV states.

**Table 1:** Age-standardized prevalences of having non-regular partners and of using female sex work in the past year among Indian men, by region and marital status, 2006.

	Not sexually active (n = 11 936)	Sexually active (n = 31 040)																
		Unmarried, married or previously married men					Unmarried men					Married men					PR for unmarried vs married men	
			≥1 NRP		≥1 FSW			≥1 NRP		≥1 FSW			≥1 NRP		≥1 FSW			
	N	N	n	(%)^a^	n	(%)^a^	N	n	(%)^a^	n	(%)^a^	N	n	(%)^a^	n	(%)^a^	≥1 NRP	≥1 FSW
**India** b																		
15–24 yrs	9737	4426	1630	(35.7)	384	(7.9)	2354	1486	(62.9)	336	(13.3)	2055	138	(6.5)	44	(1.9)		
25–34 yrs	1972	11 800	1135	(9.5)	443	(3.6)	845	459	(53.4)	141	(15.7)	10 870	656	(6.1)	289	(2.6)		
35–49 yrs	227	14 814	658	(4.4)	311	(2.1)	80	39	(45.6)	17	(19.2)	14 517	580	(4.0)	271	(1.8)		
**Total** c	**11 936**	**31 040**	**3423**	**(11.8)**	**1138**	**(3.6)**	**3279**	**1984**	**(51.5)**	**494**	**(16.9)**	**27 442**	**1374**	**(5.2)**	**604**	**(2.1)**	**9.8**	**8.0**
**(95% CI)**				**(11.4–12.2)**		**(3.4–3.9)**			**(45.6–57.3)**		**(12.8–22.0)**			**(4.9–5.6)**		**(1.9–2.3)**		
**High-HIV states**																		
15–24 yrs	1641	610	312	(52.0)	96	(16.0)	358	277	(79.3)	78	(22.2)	250	34	(13.7)	17	(6.8)		
25–34 yrs	368	2015	302	(15.5)	162	(8.5)	116	83	(73.2)	40	(35.8)	1886	215	(11.8)	119	(6.7)		
35–49 yrs	52	2638	199	(7.9)	103	(4.2)	7	5	(70.6)	2	(23.7)	2594	174	(7.1)	86	(3.6)		
**Total** c	**2061**	**5263**	**813**	**(18.4)**	**361**	**(7.8)**	**481**	**365**	**(73.1)**	**120**	**(27.8)**	**4730**	**423**	**(10.0)**	**222**	**(5.3)**	**7.3**	**5.3**
**(95% CI)**				**(17.4–19.5)**		**(7.0–8.6)**			**(54.2–86.2)**		**(15.9–43.9)**			**(9.0–11.1)**		**(4.6–6.1)**		
**Low-HIV states**																		
15–24 yrs	8096	3816	1318	(33.2)	288	(6.6)	1996	1209	(60.0)	258	(11.7)	1805	104	(5.6)	27	(1.3)		
25–34 yrs	1604	9785	833	(8.3)	281	(2.6)	729	376	(50.3)	101	(12.5)	8984	441	(5.0)	170	(1.7)		
35–49 yrs	175	12 176	459	(3.7)	208	(1.6)	73	34	(42.7)	15	(18.6)	11 923	406	(3.4)	185	(1.5)		
**Total** c	**9875**	**25 777**	**2610**	**(10.6)**	**777**	**(2.9)**	**2798**	**1619**	**(48.5)**	**374**	**(15.2)**	**22 712**	**951**	**(4.4)**	**382**	**(1.5)**	**11.1**	**9.9**
**(95% CI)**				**(10.2–11.0)**		**(2.6–3.1)**			**(42.3–54.7)**		**(10.9–20.7)**			**(4.0–4.7)**		**(1.4–1.7)**		
**PR for high-HIV vs low-HIV states**				**1.7**		**2.7**			**1.5**		**1.8**			**2.3**		**3.5**		

PR = prevalence ratio; NRP = non-regular partner; FSW = female sex worker; CI = confidence interval.

a Percentages are sample-weighted.

b Excludes the northeastern states.

c Total percentages and 95% CIs are sample-weighted and standardized to the age distribution of all 31 040 sexually active men in the study sample.

Prevalences of any NRP and of using FSW differed sharply by marital status. After standardizing for age, about 52% (1984/3279) of all unmarried men in the sample reported any NRP and about 17% (494/3279) reported FSW use; these prevalences were about 10 and 8 times as high as among married men. About 73% (365/481) of unmarried men in the high-HIV states reported any NRP and about 28% (120/481) reported using FSW. The prevalence of any NRP was highest among men aged 15–24 years in both regions, whether unmarried or married. In contrast, FSW use tended to be more prevalent at older ages. However, among married men in the high-HIV states, FSW use was most prevalent in the youngest age group.

Among all 3423 men reporting any NRP, 1138 men (age standardized prevalence 30.8%; 29.2–32.5) reported also using FSW (Table 2). The prevalence of FSW use was greater in the high-HIV states (361/813; 41.5%; 38.1–45.0) than in the low-HIV states (777/2610; 27.4%; 25.5–29.3). Unmarried men were less likely to also report FSW use than married men in both regions (high-HIV states: prevalence ratio (PR) 0.7; low-HIV states: PR 0.8). About 52% (222/423) of married men in the high-HIV states reporting any NRP also reported using FSW, compared to about 30% (382/951) of married men reporting any NRP in the low-HIV states.

**Table 2:** Age-standardized prevalence of using female sex work among Indian men reporting any non-regular partner in the past year, by region and marital status, 2006.

	Unmarried, married or previously married men			Unmarried men			Married men			PR for unmarried vs married men
		≥1 FSW			≥1 FSW			≥1 FSW		
	N	n	(%)^a^	N	n	(%)^a^	N	n	(%)^a^	
**India** b										
15–24 yrs	1630	384	(22.0)	1486	336	(21.1)	138	44	(29.6)	
25–34 yrs	1135	443	(37.7)	459	141	(29.4)	656	289	(41.7)	
35–49 yrs	658	311	(46.2)	39	17	(42.0)	580	271	(45.4)	
**Total** c	**3423**	**1138**	**(30.8)**	**1984**	**494**	**(27.2)**	**1374**	**604**	**(35.9)**	**0.8**
**(95% CI)**			**(29.2–32.5)**			**(23.7–30.9)**			**(31.5–40.6)**	
**High-HIV states**										
15–24 yrs	312	96	(30.7)	277	78	(28.1)	34	17	(50.0)	
25–34 yrs	302	162	(54.6)	83	40	(48.8)	215	119	(56.5)	
35–49 yrs	199	103	(52.7)	5	2	(33.6)	174	86	(50.9)	
**Total** c	**813**	**361**	**(41.5)**	**365**	**120**	**(35.1)**	**423**	**222**	**(52.0)**	**0.7**
**(95% CI)**			**(38.1–45.0)**			**(27.4–43.7)**			**(42.7–61.2)**	
**Low-HIV states**										
15–24 yrs	1318	288	(20.0)	1209	258	(19.5)	104	27	(23.0)	
25–34 yrs	833	281	(31.4)	376	101	(24.8)	441	170	(34.7)	
35–49 yrs	459	208	(43.3)	34	15	(43.6)	406	185	(43.0)	
**Total** c	**2610**	**777**	**(27.4)**	**1619**	**374**	**(25.3)**	**951**	**382**	**(29.9)**	**0.8**
**(95% CI)**			**(25.5–29.3)**			**(21.5–29.4)**			**(25.2–35.1)**	
**PR for high-HIV vs low-HIV states**			**1.5**			**1.4**			**1.7**	

PR = prevalence ratio; FSW = female sex worker; CI = confidence interval.

a Percentages are sample-weighted.

b Excludes the northeastern states.

c Total percentages and 95% CIs are sample-weighted and standardized to the age distribution of all 3423 men in the study sample reporting any non-regular partner in the past year.

### Risk factors for using female sex work

**Figure 1:**
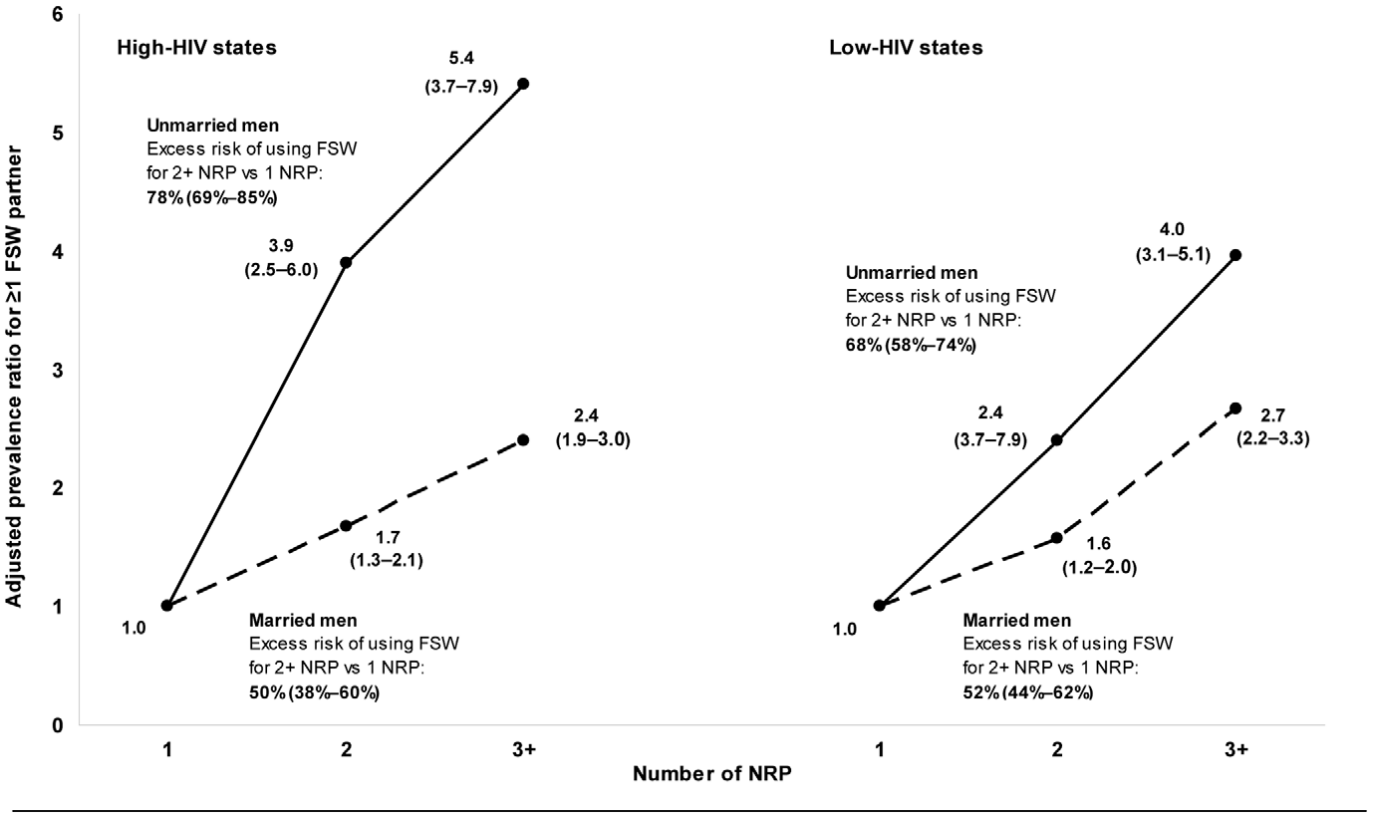
Adjusted prevalence ratios (95% CI) for use of female sex workers (FSW) comparing men with multiple non-regular partners (NRP) to men with one NRP in the past year in 2006. CI = confidence interval. All prevalence ratios (PR) are adjusted for age and education. PR for unmarried men in the high-HIV states is also adjusted for urban residency, employment in the transport sector, having heard of STI, receiving interpersonal STI/HIV/AIDS education in the past year, genital discharge or ulcer in the past year, and consistency of condom use with NRP in the past year. PR for married men in the high-HIV states is also adjusted for urban residency, employment in the transport sector, having heard of STI, receiving interpersonal STI/HIV/AIDS education in the past year, and consistency of condom use with NRP in the past year. PR for unmarried men in the low-HIV states is also adjusted for urban residency, employment in the transport sector, genital discharge or ulcer in the past year, and consistency of condom use with NRP in the past year. PR for married men in the low-HIV states is also adjusted for employment in the transport sector, awareness of a local HIV test centre, and consistency of condom use with NRP in the past year.

Tables 3 and 4 present the variables which were significantly associated with using FSW among men reporting any NRP in the high-HIV states and low-HIV states, respectively, after adjusting for other characteristics and sexual risk behaviours. Three variables were associated with FSW use across all groups of men: having more than one NRP in the past year, consistent condom use with all NRP in the past year, and being employed in the transport sector. The strongest predictor of using FSW was the number of NRP in the past year. In unmarried and married men of each region, the adjusted PR for FSW use increased with increasing number of NRP (Figure 1). Particularly notable was the PR of 5.4 (95% CI 3.7–7.9) among unmarried men reporting three or more NRP in the high-HIV states.

**Table 3:** Factors associated with using female sex work among Indian men reporting any non-regular partner in the past year in the high-HIV states, 2006.

	No FSW		≥1 FSW		Adjusted^a^ PR (95% CI)
	n	(%)	n	(%)	
**Unmarried men in the high-HIV states (n = 365)**					
*Socio-demographic characteristics*					
Residence					
Rural	142	(72.8)	55	(27.3)	1.0
Urban	103	(61.1)	65	(38.9)	1.3 (1.0–1.7)
Education					
Secondary or higher	221	(71.9)	91	(28.1)	1.0
Primary or none	24	(45.2)	29	(54.8)	1.7 (1.2–2.4)
Employed in transport sector					
No	242	(70.1)	108	(29.9)	1.0
Yes	3	(22.0)	11	(78.0)	1.8 (1.1–2.9)
*HIV/STI knowledge and awareness*					
Heard of STI other than HIV					
No	95	(60.6)	65	(39.4)	1.0
Yes	149	(74.3)	55	(25.7)	0.6 (0.5–0.8)
Interpersonal STI/HIV/AIDS education in past year					
No	158	(65.3)	88	(34.7)	1.0
Yes	87	(73.8)	32	(26.2)	0.7 (0.5–1.0)
*Sexual behaviour indicators*					
Genital discharge or ulcer in past year					
No	230	(69.6)	105	(30.4)	1.0
Yes	13	(46.9)	15	(53.2)	1.5 (1.1–2.2)
Number of NRP in past year					
1	194	(88.4)	27	(11.6)	1.0
2 or more	49	(35.9)	90	(64.2)	4.6 (3.2–6.7)
Consistent condom use with NRP in past year					
No	116	(72.7)	44	(27.3)	1.0
Yes	126	(64.9)	73	(35.1)	2.2 (1.6–3.0)
**Married men in the high-HIV states (n = 423)**					
*Socio-demographic characteristics*					
Residence					
Rural	91	(39.3)	137	(60.7)	1.0
Urban	110	(57.7)	85	(42.3)	0.8 (0.7–1.0)
Employed in transport sector					
No	185	(52.0)	157	(48.0)	1.0
Yes	16	(21.2)	65	(78.8)	1.3 (1.1–1.6)
*HIV/STI knowledge and awareness*					
Heard of STI other than HIV					
No	94	(47.4)	96	(52.6)	1.0
Yes	106	(45.9)	124	(54.1)	0.9 (0.7–1.0)
Interpersonal STI/HIV/AIDS education in past year					
No	141	(54.5)	115	(45.5)	1.0
Yes	60	(35.3)	105	(64.7)	1.3 (1.0–1.5)
*Sexual behaviour indicators*					
Number of NRP in past year					
1	136	(66.3)	64	(33.7)	1.0
2 or more	61	(27.7)	157	(72.3)	2.0 (1.6–2.5)
Consistent condom use with NRP in past year					
No	140	(63.0)	78	(37.1)	1.0
Yes	52	(27.4)	141	(72.6)	1.8 (1.5–2.2)

FSW = female sex worker; PR = prevalence ratio; NRP = non-regular partner; STI = sexually transmitted infection. Frequencies (sample-weighted percentages) for each variable exclude missing data.

a Unmarried model (model n = 352) is adjusted for age and the variables shown in the upper portion of the table; married model (model n = 399) is adjusted for age, education, consistency of condom use with spouse, and the variables shown in the lower portion of the table.

**Table 4:** Factors associated with using female sex work among Indian men reporting any non-regular partner in the past year in the low-HIV states, 2006.

	No FSW		≥1 FSW		Adjusted^a^ PR (95% CI)
	n	(%)	n	(%)	
**Unmarried men in the low-HIV states (n = 1619)**					
*Socio-demographic characteristics*					
Residence					
Rural	565	(82.7)	148	(17.3)	1.0
Urban	680	(73.8)	226	(26.3)	1.4 (1.2–1.8)
Education					
Secondary or higher	966	(81.2)	258	(18.8)	1.0
Primary or none	279	(73.2)	116	(26.8)	1.3 (1.1–1.7)
Employed in transport sector					
No	1200	(80.3)	337	(19.7)	1.0
Yes	45	(55.1)	37	(44.9)	1.6 (1.2–2.1)
*Sexual behaviour indicators*					
Genital discharge or ulcer in past year					
No	1177	(80.2)	332	(19.8)	1.0
Yes	66	(63.2)	42	(36.9)	1.7 (1.3–2.3)
Number of NRP in past year					
1	850	(89.3)	114	(10.7)	1.0
2 or more	391	(64.0)	252	(36.0)	3.1 (2.4–3.9)
Consistent condom use with NRP in past year					
No	606	(84.2)	130	(15.9)	1.0
Yes	628	(74.5)	241	(25.6)	1.5 (1.2–1.9)
**Married men in the low-HIV states (n = 951)**					
*Socio-demographic characteristics*					
Employed in transport sector					
No	519	(65.8)	302	(34.2)	1.0
Yes	49	(44.7)	80	(55.3)	1.3 (1.0–1.5)
*HIV/STI knowledge and awareness*					
Aware of local HIV test centre					
No	309	(61.2)	220	(38.8)	1.0
Yes	252	(67.2)	151	(32.8)	0.7 (0.6–0.9)
*Sexual behaviour indicators*					
Number of NRP in past year					
1	371	(75.4)	135	(24.6)	1.0
2 or more	196	(49.9)	236	(50.1)	2.1 (1.8–2.6)
Consistent condom use with NRP in past year					
No	308	(75.8)	113	(24.2)	1.0
Yes	255	(52.0)	260	(48.0)	2.0 (1.6–2.5)

FSW = female sex worker; PR = prevalence ratio; NRP = non-regular partner. Frequencies (sample-weighted percentages) for each variable exclude missing data.

a Unmarried model (model n = 1591) is adjusted for age and the variables shown in the upper portion of the table; married model (model n = 906) is adjusted for age, education, and the variables shown in the lower portion of the table.

### Absolute number of men using FSW

**Figure 2:**
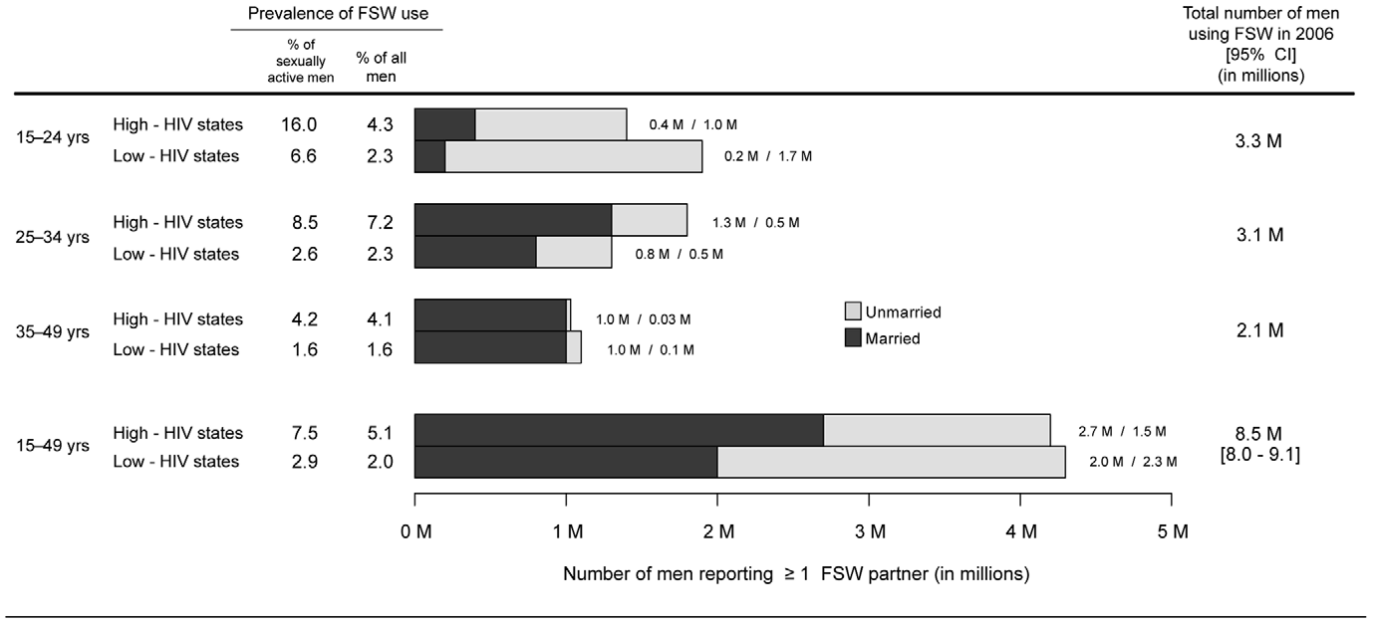
Estimated number of unmarried and married men in India reporting at least one female sex worker (FSW) partner in the past year in 2006. CI = confidence interval. Men from the seven northeastern states (i.e. Arunachal Pradesh, Manipur, Meghalaya, Mizoram, Nagaland, Sikkim and Tripura; about 4% of the national adult male population) are excluded.

The prevalences of FSW use in our study imply that about 8.5 million (95%CI 8.0–9.1) men aged 15–49 years in India used FSW in 2006 (Figure 2). Half of these men (4.2 million) were located in the high-HIV states, where nearly 8% of sexually active men were FSW clients compared to 3% of sexually active men in the low-HIV states. Nationally, more than half of all clients were married (4.7 million; 55%), with both a larger number and a larger proportion being married in the high-HIV states (2.7 million; 64%) than in the low-HIV states (2.0 million; 47%). The number of clients decreased with age in the low-HIV states, with about 44% (1.9 million) aged 15–24 years, 30% (1.3 million) aged 25–34 years, and 26% (1.1 million) aged 35–49 years. In the high-HIV states however, 33% (1.4 million) were aged 15–24 years, 43% (1.8 million) were aged 25–34 years, and 24% (1.0 million) were aged 35–49 years.

## Discussion

Our nationally representative study estimates that in 2006 more than 8 million men aged 15–49 years had at least one FSW partner in the preceding year. The observed patterns of reported FSW use confirm that most HIV transmission in India likely arises from male use of FSW [4]–[6], with onward transmission to typically monogamous wives [18].

Our results help to explain the higher HIV-prevalence in the four southern high-HIV states: men in these states were almost twice as likely to have NRP (18% vs 11%) and almost three times as likely to use FSW (8% vs 3%) as men in the low-HIV states, and half of all FSW clients in India in 2006 lived in these high-HIV states. Previous studies in the high-HIV states have identified husbands' sexual risk behaviour as the most important factor associated with wives' HIV risk [26], [27]. We found that the prevalence of marriage among sexually active men was similar in both regions, but that married men comprised a larger proportion of FSW clients in the high-HIV states than in the low-HIV states (64% vs 47%) as well as a larger absolute number of FSW clients (2.7 million vs 2.0 million).

Our results also suggest that men with multiple NRP differ from men with only one NRP mainly because they are substantially more likely to use FSW. Thus, 78% and 68% of unmarried clients with multiple NRP in the high-HIV and low HIV states, respectively, might not have used FSW had they had only one NRP. The excess risk of using FSW was lower (about 50%) among married clients with multiple NRP in both regions, reflecting that among married men, FSW use was common even among those with fewer NRP.

This study of self-reported sexual behaviour suffers some limitations. Social desirability bias, which discourages disclosure, has been observed to be more of a problem in women than in men [28]–[30]. Indeed, men, especially unmarried men, may exaggerate their NRP [31]. If unmarried men over-reported their FSW use more so than married men in our study, our estimates of the increased risk of FSW use associated with being married may be too low. The effect of such bias on our calculation of the number of FSW clients in 2006 may be limited however, given that more than half of all clients were married. Moreover, an Indian study comparing audio-assisted confidential voting interviewing to face-to-face interviewing in northern India found no difference between the two reporting modes in the proportion of young men reporting NRP or FSW [32]. Finally, we don't expect such biases to differ between the high-HIV states and low-HIV states, thus not materially affecting our regional comparisons.

While HIV prevention approaches targeted at male users of FSW may also prove effective given the social disempowerment that most FSW in India presently endure [33], the results of our study indicate that the current national strategy of peer-based condom and education interventions for FSW remains the most efficient way to curb heterosexual HIV transmission in India [34], [35]. First, the steep relationship between more NRP and higher risk of FSW use, particularly among unmarried men, suggests that few NRP are in fact “girlfriends” or non-commercial partners. Leaving aside homosexual contact (which is estimated to involve only a small proportion of men in India [18]), male and female sexual contacts in a population should be roughly comparable. Whereas about 12% of men reported NRP in India in the past year, only about 3% of women did so (7% and 2% in the high-HIV and low-HIV states, respectively; data not shown). This four-fold difference between men and women (and the large absolute number of male clients) may in part reflect under-reporting of NRP by women, but also suggests the common use of FSW, as has been shown in the United States [36]. Thus, if FSW use accounts for much of male-to-female transmission, then FSW interventions should reduce downstream HIV transmission, including transmission to wives.

Secondly, clients of FSW reported an average of 2.2 FSW partners in the preceding year (2.2 in the high-HIV states and 2.1 in the low-HIV states; data not shown). The number of FSW contacts is difficult to estimate because the frequency of contact per FSW partner was not measured. Nonetheless, 8.5 million contacts (and likely a much higher number) is a minimum estimate. Highly effective condom and education interventions are thus much more efficiently delivered by focusing on FSW rather than on their clients: about 0.36 million FSW work in the urban areas of the high-HIV states [37] compared to 4.2 million clients in urban and rural areas. (One exception is the feasibility of reaching probable FSW clients by implementing condom and education interventions among men employed in the transport sector.)

Finally, consistent condom use with all NRP in the past year was more likely among clients of FSW than among non-clients in both regions. We could not determine if higher condom use among FSW clients preceded or followed the acquisition of FSW partners, and we found few significant differences between clients and non-clients with respect to various HIV/STI knowledge and awareness indicators. Behavioural surveillance in high-risk groups in India shows that condom use in commercial sex increased between 2001 and 2006 [38]. In the high-HIV states, condom use with the last commercial partner rose from 82% to 95% among FSW and from 81% to 93% among clients. In the low-HIV states, condom use increased from 77% to 87% among FSW and from 75% to 84% among clients (data not shown). It may be that peer-based condom and education interventions for FSW have succeeded in increasing condom use despite persistent low knowledge of HIV/STI transmission, which would be consistent with African data [35].

In sum, we find a large number of men using FSW in India, particularly in the high-HIV states, and our study results argue for ensuring that condom promotion and education interventions for FSW remain a priority to curb HIV transmission in India.
